# Extraction aflatoxins from cereals using agerogel @ COF composite and evaluating ultraviolet and microwave irradiation and sonication effect on their decontamination

**DOI:** 10.1016/j.fochx.2026.103522

**Published:** 2026-01-11

**Authors:** Parvin Oroojzadeh, Mohammad Reza Afshar Mogaddam, Mohammadali Torbati, Mir Ali Farajzadeh

**Affiliations:** a Department of Food Science and Technology, Faculty of Nutrition, Tabriz University of Medical Sciences, Tabriz, Iran; b Food and Drug Safety Research Center, Pharmaceutical Sciences Institutes, Tabriz University of Medical Sciences, Tabriz, Iran; c New Material and Green Chemistry Research Center, Khazar University, 41 Mehseti Street, Baku AZ1096, Azerbaijan; d Department of Analytical Chemistry, Faculty of Chemistry, University of Tabriz, Tabriz, Iran; e Engineering Faculty, Near East University, 99138 Nicosia, North Cyprus, Mersin 10, Turkey

**Keywords:** Dispersive solid phase microextraction, Aflatoxins, Covalent-organic frameworks, Graphene aerogel

## Abstract

A dispersive solid-phase extraction method was proposed for the extraction of aflatoxins (B1, B2, G1, and G2) from cereal samples. A covalent-organic framework and graphene aerogel composite were evaluated for their capacity to extract and preconcentrate aflatoxins in the microextraction method. In the proposed method, aflatoxins were extracted with deionized water and acetonitrile (9:1, *v*/v), aflatoxins were adsorbed to a small amount (10 mg) of the sorbent, desorbed with a low volume of acetonitrile, and determined by high performance liquid chromatography-fluorescence detection. The extraction conditions including the type and amount of adsorbent, stirring conditions, concentration of NaCl, pH, and the type and volume of elution solvent optimized using a “one-variable-at-a-time” strategy. Under these optimized conditions, aflatoxins were effectively extracted from the cereal samples. Good linearity was obtained with correlation coefficients (r^2^) of 0.999, 0.996, 0.999, and 0.987 for aflatoxins B_1_, B_2_, G_1_, and G_2_, respectively. The detection limits for aflatoxins B_1_, B_2_, G_1_, and G_2_ were 0.014, 0.009, 0.021, and 0.012 μg/kg, respectively and the quantitation limits were 0.047, 0.032, 0.064, and 0.04 μg/kg respectively. The extraction recoveries of the analytes ranged from 41.7 to 52.6% using the developed method. The relative standard deviations for intra (*n* = 6) and inter-day (n = 6) precisions for aflatoxins at two concentration levels (2 and 5 μg/kg) were ≤ 8.3% and ≤ 10.2%, respectively. These results indicate that the suggested extraction protocol combined with the detection system can be applied for the sensitive analysis of trace amounts of aflatoxins in food samples.

## Highlights

• A new aerogel@covalent organic framework was synthesized, characterized and used in extraction procedure.
• DSPE of four aflatoxins were done from cereal samples
• Microwave, ultraviolet, and ultrasound-assisted degradation of aflatoxins were evaluated.
• Accurate, reliable, sensitive, and effective results were obtained by the method.

## 1 Introduction

Aflatoxins (AFs) are secondary fungal metabolites produced by Aspergillus flavus and Aspergillus parasiticus. Their most common and dangerous subtypes are AFB_1_, AFB_2_, AFG_1_ and AFG_2_ (Ramadan & Al-Ameri, 2022). The toxicity of AFs is recognized by the Food and Agriculture Organization and the World Health Organization (Frisvad, 2012; Mayer et al., 2008). Also, AFs are classified as Group 1 carcinogens by the International Agency for Research on Cancer due to their toxic, mutagenic, carcinogenic, teratogenic, and immunotoxic properties (Kumar, Gupta, Mahato, Pandhi, Pandey, Kargwal, et al., 2022). Damage to DNA, causing cancer and developmental abnormalities in embryos, and affecting the physiological condition of humans and animals are among the disadvantages of long-term exposure to AFs (Alameri, Kong, Aljaafari, Ali, Eid, Sallagi, et al., 2023; Kornsakulkarn et al., 2012). Exposure to AFs also causes acute poisoning called aflatoxicosis, which mainly leads to liver damage (Organization, W. H., and Organization, W. H., 2018; Yousif, 2025). Research has shown that the highest level of exposure to AFs-contaminated foods is observed in the children population, followed by adolescents and adults who are the least exposed group (Alameri et al., 2023; Ezekiel et al., 2021). The toxicity of AFs is typically explained by the epoxidation of the 8,9-double bond along with the greater potency related to the cyclopentenone ring of the B series as opposed to the six-membered ring of the G series (Ismail et al., 2021). Ingestion of contaminated food products such as grains, inhalation and direct contact through the skin or mucous membranes are exposure routes (Awuchi et al., 2021; Kumar et al., 2022).

Cereals and cereal-based products are among the major sources of nutrition worldwide (Tufail et al., 2023). However, contamination of these foods with AFs has raised serious concerns. Consumption of cereals and cereal-based products contaminated with AFs can lead to acute and chronic health problems related to physical and mental maturation, reproduction and the nervous system (Agriopoulou et al., 2020; Oyebamiji et al., 2024). Therefore, accurate detection methods, detoxification and management strategies for AFs in cereals and cereal-based products are crucial for food safety as well as consumer health (Oyebamiji et al., 2024).

The complexity of the food matrix eliminates extracting the effect of food matrices for accurate determination of target analytes in real samples (Hosseininezhad et al., 2023; Hu et al., 2024). Extraction methods are classified into two categories: modern and classical methods (G. Li & Chen, 2024). Nowadays, microextraction methods such as dispersive liquid-liquid microextraction and solid-phase microextraction have attracted more attention. Because microextraction methods are in line with green chemistry, the volume of organic solvents required for analyte extraction is much smaller, resulting in less waste and less toxicity. Furthermore, microextraction methods generally have excellent efficiency, short extraction time, and low cost, and can be utilized in flow systems (Jagirani & Soylak, 2022). Microextraction methods were introduced to reduce the disadvantages of traditional extraction techniques while contributing to their advantages (Peng & Cao, 2021).

In recent years, dispersive solid phase extraction (DSPE) has been used as an SPE method for the extraction, cleanup, and preconcentration of various analytes in real samples. It is a practical, simple, rapid, and low-cost technique that is associated with high extraction efficiency, high enrichment factor, and low sample solution consumption (Ghorbani et al., 2019). Covalent organic frameworks (COFs) are a class of crystalline porous materials constructed from organic monomers linked by covalent bonds. These frameworks are composed entirely of light elements such as carbon, hydrogen, oxygen, and nitrogen. Due to their highly ordered structure, COFs, like metal-organic frameworks (Kumar, 2024), exhibit remarkable properties, including high chemical stability, large surface areas, and tunable pore sizes. These features make COFs versatile materials used in various applications, including catalysis, gas storage, sensing, and separation processes. The ability to precisely control their structure allows COFs to be tailored for specific applications, offering high adsorption capacity and selective interactions with target molecules, making them particularly useful in fields like environmental monitoring and chemical sensing (J. Li et al., 2022; Wei et al., 2023). Aerogels are lightweight, highly porous materials with a unique structure. They are typically prepared by removing the liquid component from a gel through a supercritical drying process, resulting in a solid with a vast internal surface area. They can be made from various materials, including silica, carbon-based materials, polymers, and even metal-organic frameworks (Gan et al., 2025). These materials are known for their excellent thermal insulation properties, high surface area, and low density. Aerogels are widely used in applications such as energy storage, pollutant capture, heterogeneous catalysis, and gas storage. Their exceptional adsorption properties, coupled with their low weight, make them ideal for applications where both high performance and minimal weight are crucial (Bajpai et al., 2019; Martín-Illán et al., 2021). The combination of COFs and aerogels results in hybrid materials that merge the unique advantages of both. COFs contribute high surface area, tunable pore sizes, and excellent chemical stability, while aerogels offer a highly porous structure with low density and enhanced surface area. When combined, these materials exhibit superior adsorption capabilities, making them ideal for high-performance applications in fields such as DSPE and environmental monitoring. The porous aerogel structure enhances the accessibility of COFs active sites, improving the efficiency of analyte capture and desorption. Additionally, the lightweight and flexible nature of aerogels ensure that the resulting hybrid materials are easy to handle, enhancing their practical applications. Overall, the combination of COFs and aerogels leads to materials with enhanced stability, improved adsorption capacity, and tailored functionalities, making them highly suitable for advanced separation, sensing, and extraction technologies.

Since the adsorbent particles are in direct contact with the analytes in the DSPE method, the selectivity of the reaction increases, providing high extraction recovery in a short time along with clean chromatograms (Badali et al., 2023).Also, this technique uses very low amounts of adsorbent and organic solvent, supporting the green chemistry agenda and being very sensitive and economical. Obviously, to achieve a low detection limit in the DSPE method, the synthesis and use of adsorbents that show high affinity for the target analyte in the complex matrix is of great importance.

In this work, for the first time, the adsorption capability of the adsorbent synthesized from graphene oxide aerogel and COF was evaluated for the preconcentration and extraction of AFs (B_1_, B_2_, G_1_, and G_2_) from cereal samples. Among cereals, wheat flour plays an important role in the nutritional chain of humans and animals due to its high consumption worldwide. The global consumption of wheat exceeds than 740 million tons and its contamination with pollutants like AFs is a major concern (https://www.marketreportsworld.com/market-reports/wheat-flour-market-14722655, n.d). Wheat is highly susceptible to contamination by fungal toxins during storage and transportation (Jiang et al., 2022). The combination of COF and aerogel increases the adsorption capacity of the target analyte due to improved functional properties. The effect of UV and, microwaves irradiation, and sonication on reducing AFs levels was also investigated. The high adsorption capacity of COF, exceptional adsorption properties of aerogel along with high porosity, make their composite suitable for use in DSPE. The effects of different experimental parameters on the proposed DSPE method were evaluated. The analysis was performed by high performance liquid chromatography (HPLC) coupled with a fluorescence detector (FLD). The aim of this study was to develop a rapid and simple extraction method for Aflatoxins (G1, G2, B1 and B2) residues from cereal product samples (Tabriz-Iran).

## 2 Experimental

### 2.1 Reagents and materials

Analytical standards for AFs were supplied by Sigma-Aldrich (St. Louis, Missouri, USA). HPLC-grade acetonitrile (ACN) and methanol as well as analytical-reagent grade sulfuric acid, sodium nitrate, potassium permanganate, hydrochloric acid (37%, *w*/w), sodium hydroxide, ethanol, dimethylformamide (DMF), glacial acetic acid, graphite powder, ammonium citrate, terephthalaldehyde and benzidine were obtained from Merck (Darmstadt, Germany). A mixed working stock solution of AFs (500 ng/mL each) was prepared in methanol and stored in a refrigerator at 4 °C.

### 2.2 Instrumentation

Separation and quantification of the analytes were performed using an Agilent liquid chromatograph (Model, 1200; Agilent Technologies; USA) equipped with a degasser, quaternary pump, and FLD. The separation took place on an Eclipse EDB octadecyl silane analytical column (Agilent Technologies) with a column length of 15 cm, internal diameter of 4.6 mm, and particle size of 5 μm.The column was kept at 35 °C in an oven during analysis. The mobile phase was prepared by mixing methanol, ACN, and 1% acetic acid in water and flowed at 1.0 mL/min. The mobile phase composition was changed during analysis according to Table S1. The excitation and emission wavelengths of FLD were set at 360 and 430 nm, respectively. Ultrasound treatment was carried out using a sonic probe (Sonic, Dismembrator, model Quigley-Rochester, Rochester, NY, USA) equipped with a 1.25 cm titanium probe at frequencies ranging from minimum to maximum kHz. A laminar microbial hood (Fater, model LH608, Tehran, Iran), with a UV lamp at a fixed wavelength of 254 nm was utilized. A microwave oven, (Samsung, model GE401, Malaysia) with a power range of 100–900 W was also employed. The surface morphology and structure of the sorbent were exmained using a scanning electron microscope (SEM, Tescan, Czech Republic). X-ray diffraction patterns (XRD, Siemens, Germany) and Fourier Transform Infrared spectroscopy (FTIR, Brucker, Germany) analysis were performed on the sorbent.

### 2.3 Real samples preparation

Cereal product samples including of three types of wheat flour used for baking Lavash, Sangak, and Barbari, as well as six different types of bread (Cornbread, Barbari bread, Ahari bread, Barley bread, Rice bran bread, and sweet bread), were obtained from local groceries and bakeries in East Azarbaijan Province (Tabriz, Iran). The bread samples were left to dry at room temperature and then ground. Afterwards, 1 g of each sample was taken and analyzed both before and after being spiked with the AFs. Additional flour and bread samples were taken after analysis by a previously published method to confirm the absence of target AFs and were utilized as blank samples. The spiked samples were allowed to stand for 15 min to ensure complete penetration of the analytes, after which 0.5 mL of ACN and 1.5 mL of deionized water were added. The mixture was then vortexed for 5 min. Following that, centrifugation was performed to separate the liquid phase. The supernatant was transferred into a clean falcon tube for subsequent analytical procedures and diluted with deionized water up to 5 mL.

### 2.4 Synthesis of aerogel@COF adsorbent

(a) Preparation of graphene oxide (GO): To prepare GO from graphite powder, a modified Hummer method was used. In this process, 1 g of graphite powder and 0.5 g of sodium nitrate were combined, then 23 mL of sulfuric acid was added while stirring. After 1 h, 3 g of potassium permanganate was slowly added to the solution. Throughout this stage, the temperature was controlled to ensure it stayed below 20 °C to prevent overheating and potential explosions. Following 12 h of stirring at 35 °C, the resulting mixture was diluted by adding 500 mL of water and *v*igorously stirring. To confirm the completion of the reaction with potassium permanganate, the suspension was treated with 5 mL of a 30% *w*/w H_2_O_2_ solution. The resulting mixture was then filtered, washed with HCl (1 mol/L) and deionized water, and finally dried in an oven adjusted to 80 °C for 4 h (Shahriary & Athawale, 2014).

(b) Preparation of aerogel: First, 75 mg of ammonium citrate was added to GO (3 mg dispersed in 5 mL deionized water) and sonicated for 5 min. Then the pH of the suspension was regulated to 1 with an HCl solution (2 mol/L). Afterward, this mixture was filled into a hydrothermal Teflon-lined *v*essel and placed in an oven adjusted at 120 ± 5 °C. After nine hours, the prepared gel was eluted with deionized water to remove the excess acid and immersed into an ethanol solution (20%, *v*/v) for 48 h, and finally freeze-dried to obtain the final product (Kang et al., 2021).

(c) Synthesis of COF: in order to prepare pure COF, 400 mg of terephthalaldehyde was mixed with 550 mg of benzidine dissolved in DMF (20 mL). This mixture was sonicated for 5 min and 2 mL of glacial acetic acid solution at a concentration of 0.5 mol/L was added into it over 1 h. During the addition of the acetic acid solution, the mixture was stirred continuously at 300 rpm. The yellow particles were collected by centrifugation and washed with an ethanol solution (50% v/v) five times and then dried in an oven adjusted to 80 °C (Okhravi et al., 2024).

(d) Preparation of aerogel@COF composite: First 225 mg of the synthesized aerogel was dispersed into 20 mL of DMF (via sonication for 30 min) and 0.4 g of terephthalaldehyde and 0.55 g of benzidine were added to this mixture. After 5 min of sonication, 2 mL of glacial acetic acid was added to the solution over 1 h with rotation. After filtration, the residual product on the filter paper was washed with deionized water and ethanol and dried to obtain the final product.

### 2.5 Sample pretreatment procedure

The supernatant obtained from the samples according to Section 2.3 or spiked deionized water with the AFs at a concentration of 25 μg/kg (each analyte) was transferred into a 15-mL glass test tube. Sodium chloride (0.25 g) was dissolved in the solution and then 10 mg of the prepared aerogel@COF was added to the solution and vortexed for 1 min. Centrifugation was performed to separate the supernatant. The supernatant was discarded and 50 μL of ACN was added to the solid phase collected at the bottom of the falcon. Then, it was subjected to sonication for 2 min. After centrifugation, the supernatant was separated, and 1 μL of it was injected into the HPLC-FLD for analysis. The steps of this procedure are shown in Fig. S1.

### 2.6 Statistical analysis

Data processing was conducted using the computer program Excel 2016 (Microsoft Office®). The results from three independent experimental studies were expressed as mean ± standard deviation.

## 3 Results and discussions

### 3.1 Characterization of sorbent

Fig. S2a displays the FTIR spectra of aerogel, aerogel@COF (25:75%), and COF samples. In the aerogel, the peak at the wavenumber of 3435 cm^−1^ is attributed to the presence of hydroxyl functional groups (O—H). C=O, and C=C bonding peaks are weakly observed at 1716 cm^−1^, and 1568 cm^−1^, respectively. The peak of the C—O bond is observed at 1167 cm^−1^ (Ahmadi et al., 2021). For the aerogel@COF (25:75%), the O—H bond peak appear with lower intensity at the wavenumber of 3447 cm^−1^. Low intensity C—H bond peaks are detected at 2922 cm^−1^ and 2864 cm^−1^. C=O, and C=C bonds are present at 1693 cm^−1^, and 1481 cm^−1^, respectively. The C—O bond peak is at the wavenumber of 1191 cm^−1^. For the COF, O—H, C—H, C=O, C=C, and C—O bonding peaks are present at the wavenumbers of 3452 cm^−1^, 2865 cm^−1^, 1693 cm^−1^, 1483 cm^−1^, and 1192 cm^−1^, respectively. Comparing these spectra shows that the intensity of peaks at wavenumbers of 1693 cm^−1^, 1192 cm^−1^, 836 cm^−1^, and 572 cm^−1^ originating from the COF were reduced by the formation of the composite, which decreased its contribution in the mixture. It is evident that the broad peak originating from the aerogel was also presented in the composite. Fig. S2b exhibits the XRD patterns of aerogel, aerogel@COF (25: 75%), and COF. In the prepared aerogel, a broad peak is observed around 2θ of 26°. This might be due to the porous and amorphous structure of the prepared aerogel, as amorphous structures usually show broad peaks, while crystalline structures often display sharp peaks. The XRD patterns of the aerogel@COF (25: 75, %) was at 2θ of 20°, 2θ of 23°, and 2θ of 28°. The main peaks for the COF were at 2θ of 20°, 24°, and 29°, respectively. To study the morphology of the samples, SEM analysis was performed. Fig. S3 depicts the SEM images of aerogel, aerogel@COF (25: 75, %), and COF. As depicted in Fig. S3a, the layered, porous and non-uniform structure of the investigated aerogel is visible. This structure is suitable for adsorbing purposes, since the available inner and outer surfaces are abundant in aerogels. The average particle size of aerogel is around 34.30 nm. Fig. S3b prove the porous netted structure with a high surface area of aerogel@COF (25: 75, %), while the average particle size of this sample is 28.45 nm. Fig. S3c illustrates the layered and porous structure of the COF sample with cracks on its surface and an average particle size of 34.97 nm, which is favorable for adsorbing (Hu et al., 2022; Jiang et al., 2022; Keshavarz et al., 2021).

### 3.2 Optimization of extraction procedure

#### 3.2.1 Selection of adsorbent

**Fig. 1:**
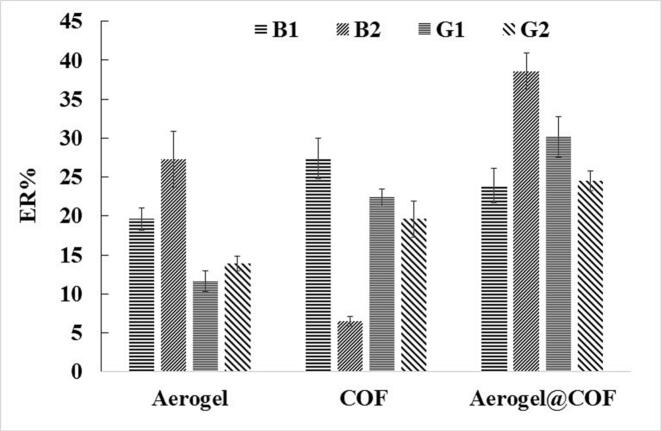
Selection of sorbent type. Conditions: sample, 5 mL deionized water spiked with AFs at a concentration of 5 ng/mL; sorbent amount, 20 mg; agitation type (time) in adsorption step, vortexing (2 min); elution solvent type (volume), ACN (50 μL); and stirring type (time) in desorption step, vortexing (2 min).

Various extraction techniques are available for sample preparation, each tailored to specific analytes and matrix types. However, after most extraction methods, the target analytes are usually recovered in either an aqueous or organic solution, requiring additional concentration or clean-up steps. In this study, a DSPE approach was tested as an alternative to the traditional solid phase extraction cartridge format for AFs. Given the significance of sorbent type in illustrating the efficiency of the prepared composite, three separate experiments were done using aerogel, COF, and aerogel@COF (50:50, %) composite. In these trials, 20 mg of each compound was used for extracting of target AFs from 5 mL of deionized water spiked with the analytes (5 ng/mL each). The results shown in Fig. 1, indicate that the extraction recovery (ER) of aerogel and COF are significantly lower than aerogel@COF composite. The enhanced extraction efficiency of aerogel@COF composite compared to pristine aerogel or COF is related to the synergistic effects of their individual properties providing high porosity, a large surface area, a good mass transfer rate, and specific chemical functionalities. So, the aerogel@COF composite was opted as the sorbent for the other tests.

#### 3.2.2 Optimization of composite components ratio

**Fig. 2:**
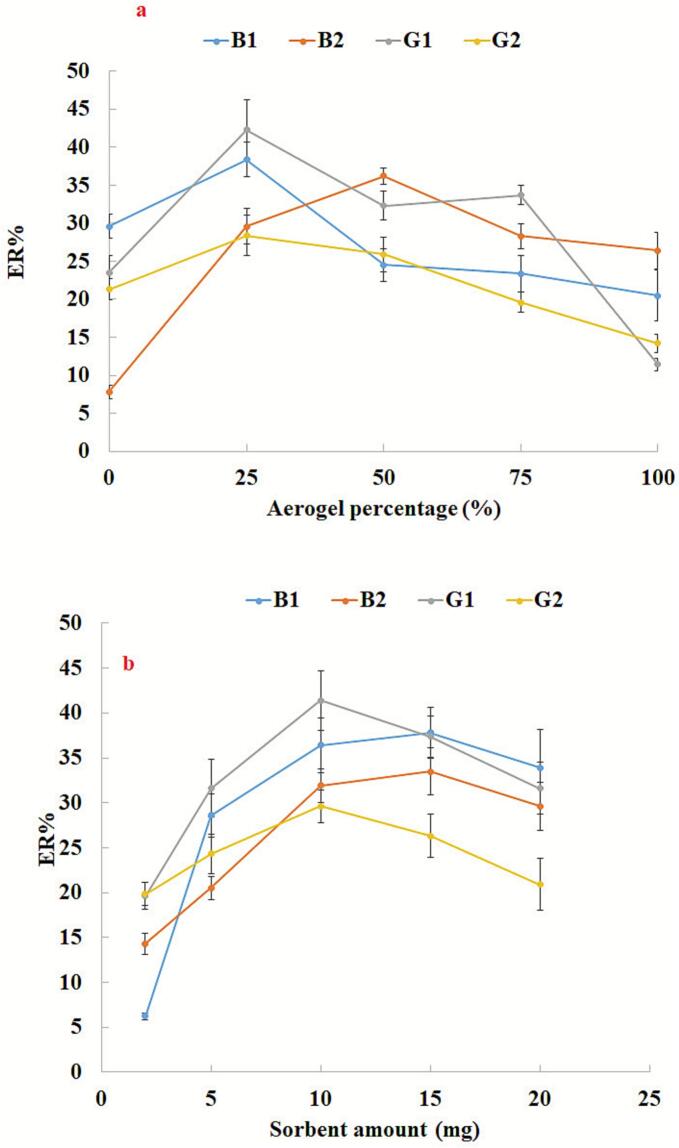
Optimization of sorbent composition and amount. (a) Conditions: are the same as those use in Fig. 1, except aerogel@COF composite was used as the sorbent. (b) Conditions: are the same as those use in Fig. 2a, except aerogel@COF composite made from 25% of aerogel was chosen as the sorbent.

The previous step confirmed the synergistic effect of aerogel and COF in the composite that was prepared. Thus, the percentage of these components can impact the efficiency of the method. Because of this, the molar ratio of aerogel to COF used in the preparation of the composite needs to be optimized. This parameter was tested by preparing aerogel and COF composites with varying amounts of these components and testing their effectiveness in extracting AFs, independently. The results of these tests are shown in Fig. 2a. Among the materials tested, the composite made from 25% aerogel and 75% COF was effective for all AFs except for AFB1. The decrease in method efficiency with increased aerogel content may be due to inadequate desorption of the analytes from the composite particles or incomplete dispersion in the sample solution. Therefore, the next tests were performed using the composite prepared from 25% aerogel and 75% COF.

#### 3.2.3 Optimization of sorbent amount

The analytes present in the grain sample can be extracted by a suitable adsorbent during the DSPE step. The amount of adsorbent plays an important role in the efficiency of the method due to its direct role in providing adsorption sites. It is expected that higher amounts of sorbent provide effective extraction of the analytes, but it is not a solid fact due to the need for analytes to be eluted by higher volumes of eluent. In this method, aerogel@COF nanoparticles were used for the extraction of analytes. By using aerogel@COF nanoparticles with high porosity and strong adsorption capacity, this method is efficient for the extraction of analytes.Different amounts of aerogel@COF nanoparticles (2, 5, 10, 15, and 20 mg) were used for the extraction of analytes. These values were selected based on our preliminary experiments, taking into account the lowest possible usage of adsorbent while still maintaining its high adsorption capacity. The results (Fig. 2b) showed that the amount of 10 mg of the adsorbent is more efficient among the other amounts tested. Decreasing the analytical data at the amount much than 10 mg is related to the incomplete desorption of the analytes from sorbent surface or limited contact area of the adsorbent. Therefore, 10 mg was selected for further experiments.

#### 3.2.4 Stirring type and time in adsorption step

The efficiency of the DSPE method can be greatly influenced by the type of agitation used to mix the analyte solution with the adsorbent. To optimize ER, both sonication and vortexing were tested. As shown in the results (Fig. S4a), vortexing proves to be more effective than sonication in extracting the analytes. This improvement is likely due to the enhanced dispersion of the sorbent in the solution achieved through vortexing. Consequently, vortexing was selected for use in further experiments.

The optimization of adsorption time is a critical parameter in the DSPE procedure, as insufficient contact time can result in incomplete extraction of analytes. To establish the optimal adsorption duration, a series of experiments was conducted by varying vortexing time from 1 to 5 min in one-min increments. The experimental data (Fig. S4b) indicated that a *v*ortexing time of 1 min provides the most efficient adsorption performance.

#### 3.2.5 pH study

The pH of the solution used in DSPE, as the aqueous phase, can significantly impact the efficiency of the method due to its effect on the interaction of the analytes with the adsorbent and the sorbent stability. To in*v*estigate the role of aqueous phase pH in the method efficiency, several experiments were done on the solutions adjusted within the range of 2–12. The data (Fig. S5) illustrated that ERs were nearly constant for the analytes across the studied pH range confirming the good stability of both the sorbent and analytes at various pH levels. As a result, there was no need for pH adjustment.

#### 3.2.6 Salt addition

The addition of salt to the aqueous solution can enhance the adsorption of analytes onto the sorbent by inducing a salting-out effect, which decreases the solubility of the analytes in the aqueous solution. This phenomenon can thereby improve the extraction efficiency of the method. To investigate the influence of ionic strength, sodium chloride was added to the solution at concentrations ranging from 0 to 20% (*w*/*v*). The extraction procedure was then applied to each condition. The results (Fig. S6) indicate a positive correlation between salt concentration and extraction efficiency, with the maximum enhancement observed at 5% NaCl for all AFs, except AFG2. Therefore, 5% w/v salt concentration was selected for all subsequent analyses.

#### 3.2.7 Selection of elution solvent type and volume

**Fig. 3:**
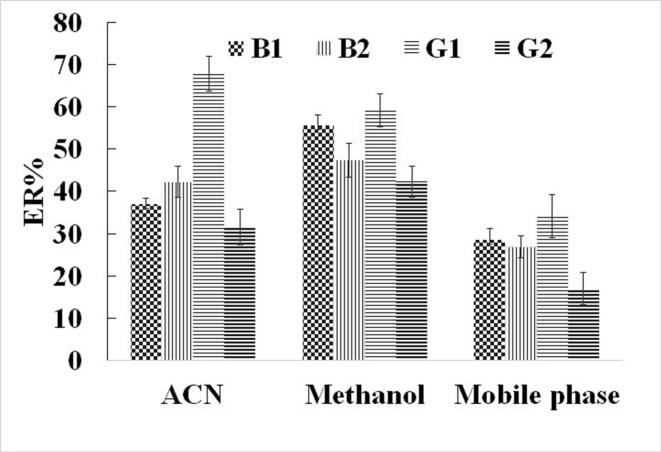
Selection of elution solvent. Conditions: are the same as those use in Fig. 2, except 5% *w*/*v* NaCl was dissolved in the aqueous solution. Also, 10 mg was the sorbent amount and 1 min of vortexing was the agitation in adsorption step.

In DSPE, once the analytes are captured by the absorbent, they must be effectively released from its surface. This is typically achieved using an appropriate organic or aqueous solvent. The choice of elution solvent significantly impacts the method's efficiency, as analyte solubility varies across different solvents. To determine the most effective solvent, the adsorbed analytes onto the sorbent surface were eluted using methanol, mobile phase, and ACN. As illustrated in Fig. 3, ACN produces the strongest analytical signals for all AFs as the effective eluate of the analytes from sorbent particles. Therefore, ACN was chosen for the subsequent procedures.

The selected eluent must be used in the optimal volume to ensure complete desorption of the analytes while minimizing consumption, thus enhancing the method's efficiency and environmental friendliness. In this method, the volume of ACN was changed from 50 to 150 μL to determine the optimal volume. The results (Fig. S7) showed that the lowest value of ACN tested in these experiments can elute the analytes similarly to higher volumes. Thus, 50 μL of ACN was chosen as the optimal amount.

#### 3.2.8 Stirring type and time in desorption step

In the DSPE process, selecting an appropriate agitation method and duration is crucial for effectively desorbing analytes from the adsorbent's active sites, thus improving removal efficiency and overall extraction performance. During the procedure, a mixture of elution solvent (ACN) and sorbent particles was subjected to either sonication or vortexing at the same time. As demonstrated Fig. S8a, sonication was found to be more effective than vortexing under these conditions.

Sonication facilitated in the quick desorption of analytes from the surface of aerogel@COF nanoparticles by enhancing the contact area between the elution solvent and nanoparticles, thereby enhancing analyte desorption and the method's efficiency. To evaluate the impact of sonication time on method's performance, the mixture of nanoparticles and elution solvent was sonicated for intervals ranging from 1 to 5 min. The results (Fig. S8b) indicate that the method's efficiency increases up to 2 min and then levels off. It is notable that desorption times shorter than 1 min were also tested and the results revealing poor repeatability in the results. Therefore, a sonication time of 2 min was selected for the subsequent steps.

### 3.3 Optimization AFs extraction from cereal sample

#### 3.3.1 Selection of aqueous solution composition

In the research, an optimized method will be used to extract AFs from various cereal samples. The limited application of DSPE on solid samples like cereals results in the extraction of target compounds into an aqueous solution. The composition of the aqueous solution must be such that it can extract all analytes from the sample matrix. Various aqueous solutions were prepared by mixing 1 mL of deionized water and 4 mL of organic solvents including ACN, propanol, acetone, and ethanol. These solutions were used for the extraction of AFs from wheat flour samples (as the candidate from cereals) spiked with the AFs at a concentration of 5 μg/kg (each analyte). These solutions were preconcentrated by the DSPE procedure. Additionally with these experiments 5 mL of deionized water spiked with the analytes at the same concentration was also extracted by the method and their results were shown in Fig. S9a. The experimental results depicted that the ACN and deionized water mixture is a more appropriate solvent among the other mixtures and it has comparable results with the solution made from deionized water. The decrease in method ER% in the presence of ACN compared to deionized water can be related to the increasing solubility of the analytes in the aqueous solution, limiting their migration coefficient.

#### 3.3.2 Optimization of aqueous solution volume

The volume of ACN in the mixture of ACN and deionized water is the most significant factor affecting the extraction of AFs from cereal samples. It affects the solubility of analytes in the aqueous solution, and increasing its volume is expected to have a positive effect on transferring analytes into the aqueous phase. However, it also affects the efficiency of the subsequent DSPE step by reducing analyte interactions with the sorbent and increasing their solubility. In this step, various mixtures of ACN and deionized water were prepared by combining different volumes of ACN in the range of 0.5–1.5 mL. The resulting data (Fig. S9b) showed that increasing the ACN volume decreases the method efficiency. To obtain the highest ER%, 0.5 mL was chosen to be used in the next steps.

### 3.4 Method validation

The validation procedure for the method involved using spiked cereal samples (blank wheat flour as the candidate for all samples) with the analytes. The sample was spiked with the analytes at concentrations of 0.1, 0.5, 1.0, 2.0, 5.0, 10, 25, and 50 μg/kg each. The collected data demonstrated that the method was linear within the concentration range of 0.064–50 μg/kg for the analytes. The coefficients of determination (r^2^) ≥0.987. Additionally, limits of detection (LODs: signal (S)/noise (N) = 3), and limits of quantification (LOQs: S/N = 10) were obtained in the range of 0.009–0.021 μg/kg and 0.032–0.064 μg/kg for the analytes, respectively. The repeatability of the method was determined to be acceptable, with the relative standard deviations (RSD) for inter-(*n* = 6) and intra-day (n = 6) precisions for AFs at two concentration levels (2 and 5 μg/kg each) being ≤9% and ≤ 11%, respectively. The ERs of analytes ranged from 41.7 to 52.6% using the developed method. To evaluate of the method's accuracy, blank wheat flour was spiked with AFs at concentrations of 2 and 5 μg/kg each analyte, and their concentrations were determined after the method was performed. The results confirmed that the deviation of concentrations was in the range of −6% to +11%. The data were summarized in Table S2.

### 3.5 Analysis of cereal samples

To verify the feasibility of the proposed approach in the analysis of target AFs (B1, B2, G1, and G2) in cereal samples, the method was performed on nine different sample (as detailed in Section 2.3) After extraction and analysis of the samples, it was concluded that all samples were free of the target analytes except for AFB1, which was found in the rice bran sample at a concentration of 1.6 ± 0.09 μg/kg. To investigate the matrix effect, all the studied samples along with the control samples were spiked with the analytes at concentration levels of 2 and 5 μg/kg of each AF. The samples were then extracted using the proposed method and analyzed using HPLC-FLD. The results, expressed as mean relative recoveries (calculated by dividing the concentration found in the spiked sample by the concentration found in a spiked control samples with the same concentration then multiplying by 100) are summarized in Table 1. Relative recoveries in the range of 84–105% indicate a negligible matrix effect of the samples evaluated.

**Table 1:** Assay of results (data are mean relative recovery ± standard deviation of three repeated analysis) for matrix effect of tested samples.

Analyte	Wheat flour used for baking of			Bread samples					
	Lavash	Sangak	Barbari	Cornbread	Barbari bread	Ahari bread	Barley bread	Rice bran	Sweet bread
All samples were spiked at a concentration of 2 μg/kg of each analyte									
AF B1	94 ± 3	87 ± 6	96 ± 5	97 ± 3	92 ± 3	92 ± 4	90 ± 5	98 ± 3	91 ± 3
AF B2	88 ± 5	105 ± 6	97 ± 6	98 ± 4	96 ± 3	89 ± 3	90 ± 6	93 ± 2	93 ± 5
AF G1	98 ± 4	101 ± 5	98 ± 4	96 ± 3	98 ± 5	99 ± 8	92 ± 3	91 ± 6	100 ± 3
AF G2	88 ± 5	97 ± 3	99 ± 3	99 ± 5	92 ± 4	98 ± 4	97 ± 5	98 ± 2	100 ± 5
All samples were spiked at a concentration of 5 μg/kg of each analyte									
AF B1	89 ± 2	84 ± 5	91 ± 3	91 ± 4	98 ± 4	91 ± 6	94 ± 3	92 ± 4	98 ± 8
AF B2	101 ± 3	98 ± 3	91 ± 3	92 ± 4	97 ± 5	94 ± 5	98 ± 5	97 ± 5	93 ± 2
AF G1	105 ± 6	96 ± 3	92 ± 5	92 ± 7	93 ± 4	96 ± 2	98 ± 5	94 ± 3	99 ± 4
AF G2	92 ± 4	91 ± 2	97 ± 5	93 ± 4	101 ± 6	92 ± 5	94 ± 3	99 ± 1	100 ± 5

Table 1.

### 3.6 Elimination of AFs content of samples

The demand for a non-chemical approach to eliminate AFs in cereals is high in order to prevent cereal wastage. Among the non-chemical approaches, UV light, sonication, and microwave exposure are the most accessible choices. This study investigated, the effects of microwave exposure, UV light, and ultrasonication to determine the most effective experimental conditions for decontaminating AFs. The experiments were done on four types of samples consisting of wheat flour, rice bran bread, barley bread, and cornbread. Initially, the effect of UV light on the concentration of AFs was investigated by exposing 100 g of each sample spiked with analytes at a concentration of 10 μg/kg each to UV light for 30 min. The analyte concentration before and after UV exposure was measured and summarized in Table 2, showing a decrease in analyte levels ranging from 6.8% to 28%.

**Table 2:** Alteration of the analyte's concentration in the various samples after and before placing under UV light.

	Concentration of the analytes after extraction by the method in															
Sample	Wheat flour for baking Lavash bread				Cornbread				Barely bread				Rice bran bread			
Analyte	B1	B2	G1	G2	B1	B2	G1	G2	B1	B2	G1	G2	B1	B2	G1	G2
Without UV	9.4 ± 0.6	9.7 ± 0.6	10.3 ± 0.4	9.5 ± 0.8	10.4 ± 0.2	9.9 ± 0.4	9.8 ± 0.5	9.8 ± 0.5	10.8 ± 0.5	10.3 ± 0.5	9.4 ± 0.2	9.7 ± 0.2	10.9 ± 0.4	10.8 ± 0.3	9.1 ± 0.4	10.6 ± 0.3
After UV for 30 min	8.2 ± 0.2	9.1 ± 0.3	7.4 ± 0.3	8.0 ± 0.2	8.5 ± 0.1	9.0 ± 0.2	7.6 ± 0.6	8.4 ± 0.4	9.3 ± 0.3	9.6 ± 0.6	8.8 ± 0.2	8.7 ± 0.5	9.6 ± 0.3	9.3 ± 0.4	8.2 ± 0.3	8.5 ± 0.3
Reducing rate (%)	13	7.8	28	15	18	10	22	17	13	7.1	6.8	10	12	14	10	20

**Fig. 4:**
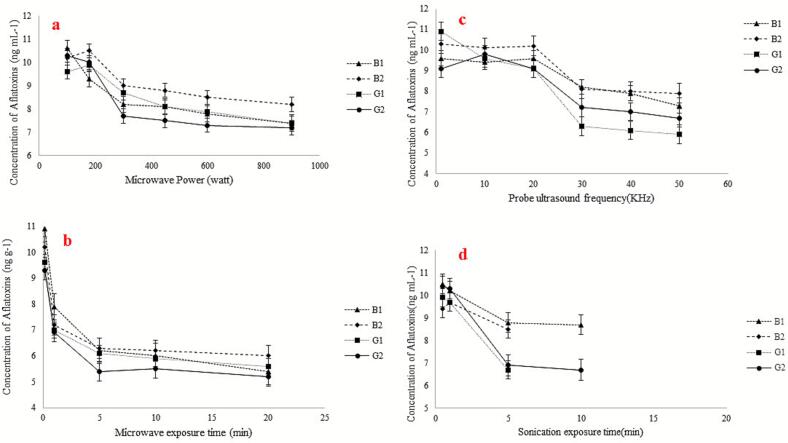
Selection of microwave exposure power and time and sonication frequency and time on the AFs concentration. Conditions: are the same as those use in Fig. 3, except 50 μL of ACN was used as the elution solvent.

The effect of microwave exposure on the elimination of AFs was investigated. Various experiments were conducted with different powers ranging from 100 to 900 W to determine the optimal microwave power. The results (Fig. 4a) indicated a decrease in analyte levels with 300 W identified as the optimal microwave power. After determining the optimal microwave power, exposure times ranging from 0.16 min (10 s) to 30 min were tested. The results (Fig. 4b) showed a decrease in analyte levels with increasing exposure time, with 5 min selected as the optimal microwave exposure time. The optimized microwave conditions were then applied to various cereal samples resulting in a reduction in analyte levels ranging from 10% to 45% (Table 3).

**Table 3:** Alteration of the AFs concentration in the various samples after and before placing under microwave exposure.

	Concentration of the analytes after extraction by the method in															
Sample	Wheat flour for baking Lavash bread				Cornbread				Barely bread				Rice bran bread			
Analyte	B1	B2	G1	G2	B1	B2	G1	G2	B1	B2	G1	G2	B1	B2	G1	G2
Without microwave exposure	9.9 ± 0.1	9.4 ± 0.5	10.0 ± 0.3	9.8 ± 0.2	10.6 ± 0.6	10.3 ± 0.4	9.3 ± 0.4	9.5 ± 0.7	10.2 ± 0.4	9.4 ± 0.3	9.8 ± 0.4	9.3 ± 0.6	9.3 ± 0.23	10.1 ± 0.4	10.5 ± 0.6	10.1 ± 0.5
Aftermicrowave exposure	6.2 ± 0.5	6.3 ± 0.2	6.1 ± 0.1	5.4 ± 0.5	8.2 ± 0.4	7.6 ± 0.4	7.7 ± 0.3	8.5 ± 0.4	6.2 ± 0.2	5.3 ± 0.5	7.6 ± 0.6	5.3 ± 0.4	7.2 ± 0.3	7.9 ± 0.9	8.5 ± 0.4	8.3 ± 0.9
Reducing rate (%)	37	31	39	44	23	26	17	10	40	43	22	45	22	21	19	17

Furthermore, the effect of sonication on the removal of AFs was investigated by optimizing sonication frequency and time within the ranges of 1–50 kHz and 0.5–20 min, respectively. The results (Fig. 4c and d) showed a significant decrease in analyte levels at 30 kHz and 5 min. These optimized values were the used on various samples spiked with analytes at a concentration of 10 μg/kg of each AF, resulting in a reduction in AF levels ranging from 12% to 41% (Table 4).

**Table 4:** Alteration of the AFs concentration in the various samples after and before placing under sonication.

	Concentration of the analytes after extraction by the method in															
Sample	Wheat flour for baking Lavash bread				Cornbread				Barely bread				Rice bran bread			
Analyte	B1	B2	G1	G2	B1	B2	G1	G2	B1	B2	G1	G2	B1	B2	G1	G2
Without sonication	10.2 ± 0.3	9.7 ± 0.6	9.7 ± 0.5	10.3 ± 0.4	10.8 ± 0.4	10.2 ± 0.4	9.7 ± 0.5	9.4 ± 0.3	9.3 ± 0.6	9.3 ± 0.7	10.8 ± 0.7	10.9 ± 0.6	9.6 ± 0.2	9.7 ± 0.5	9.8 ± 0.4	9.9 ± 0.5
After sonication	8.8 ± 0.5	8.5 ± 0.3	6.7 ± 0.5	6.9 ± 0.5	8.7 ± 0.2	6.3 ± 0.2	6.1 ± 0.3	6.8 ± 0.5	8.4 ± 0.2	7.6 ± 0.4	6.3 ± 0.4	7.2 ± 0.3	7.8 ± 0.6	6.1 ± 0.6	6.8 ± 0.5	7.8 ± 0.7
Reducing rate (%)	13	12	30	33	19	38	37	27	10	19	41	33	18	37	30	21

### 3.7 Comparison of the method with other approaches

The method used in this study is a new technique developed in line with the principles of green chemistry and the reduction of organic solvents. The results of comparing the proposed method with previous methods on various samples including milk, pistachios, peanut oil, and wheat in terms of RSD, LOD, LOQ and linear ranges (LRs) of the calibration curves are reported in Table S3. The high sensitivity of the method, wide LRs and acceptable RSD% compared to the other methods indicate the efficiency of the proposed method. The values obtained for LOD and LOQ of the offered method are lower than other methods except the method performed by DSPE–HPLC–FLD in milk sample. These researchers used on-line photo-chemical derivatization to enhance the detection of aflatoxin B_1_ (Shuib & Saad, 2022). Low values in LOD and LOQ indicate the sensitivity of the proposed method. The LRs of the proposed method are also broader than other methods. Also, the RSD% of the proposed method is either better or comparable to other methods, indicating the appropriate reproducibility of the proposed method.

## 4 Conclusions

In this research, a new method was proposed involving the use of aerogel@COF adsorbent coupled with DSPE for the effective preconcentration of AFs in cereal samples. The prepared adsorbent demonstrated high capability for the extraction of AFs under optimized DSPE conditions. The combination of COF and aerogel composites enhanced the adsorbent stability, increased surface area and provided more active sites for analyte adsorption. The use of DSPE aligns with green chemistry principles, by reducing the need for organic solvents and chemicals, making it economically viable. DSPE, as a microextraction technique, reduces the use of solvents, minimizes waste generation, and can be coupled with other green analytical chemistry approaches. Low LODs (0.009–0.021 μg/kg) and LOQs (0.032–0.064 μg/kg), wide LRs (0.064–50 μg/kg), high ERs (42–53), and good repeatability (RSD ≤ 10.1) can be considered as the main advantages of the proposed method. Thus, the proposed extraction method combined with HPLC-FLD could be applied for sensitive analysis of trace AFs in cereal samples. Also, by exposing cereal samples to ultraviolet light, microwave exposure, and sonication can reduce the amount of aflatoxins by ≤28%, ≤ 45%, and ≤ 41%, respectively, indicating that the use of these technologies is effective in reducing toxins.

## CRediT authorship contribution statement

**Parvin Oroojzadeh:** Writing – original draft, Validation, Methodology, Investigation, Formal analysis, Conceptualization. **Mohammad Reza Afshar Mogaddam:** Writing – original draft, Validation, Methodology, Formal analysis, Data curation, Conceptualization. **Mohammadali Torbati:** Writing – original draft, Methodology, Investigation, Formal analysis, Conceptualization. **Mir Ali Farajzadeh:** Writing – original draft, Validation, Methodology, Investigation, Conceptualization.

## Declaration of competing interest

The authors declare that they have no known competing financial interests or personal relationships that could have appeared to influence the work reported in this paper.

## Data Availability

The data that has been used is confidential.

## Appendix A Supplementary data

Supplementary material Image 1
