# Supplementary material for: Extraction aflatoxins from cereals using agerogel @ COF composite and evaluating ultraviolet and microwave irradiation and sonication effect on their decontamination
Source: Food Chem X. 2026 Jan 11;34:103522. doi: 10.1016/j.fochx.2026.103522 (PMC12874583; doi:10.1016/j.fochx.2026.103522)
Supplement: Supplementary file 1 — Supplementary material [file mmc1.docx]

**Supplementary data**

**Extraction aflatoxins from cereals using agerogel @ COF composite and evaluating ultraviolet and microwave irradiation and sonication effect on their decontamination**

Parvin Oroojzadeh^1^, Mohammad Reza Afshar Mogaddam^2,3^, Mohammadali Torbati^1,*^ Mir Ali Farajzadeh^4,5^

*^1^Department of Food Science and Technology, Faculty of Nutrition, Tabriz University of Medical Sciences, Tabriz, Iran*

*^2^Food and Drug Safety Research Center, Pharmaceutical Sciences Institutes, Tabriz University of Medical Sciences, Tabriz, Iran*

*^3^ New Material and Green Chemistry Research Center, Khazar University, 41 Mehseti Street, Baku AZ1096, Azerbaijan*

*^4^ Department of Analytical Chemistry, Faculty of Chemistry, University of Tabriz, Tabriz, Iran*

*^5^Engineering Faculty, Near East University, 99138 Nicosia, North Cyprus, Mersin 10, Turkey*

*Corresponding author: Dr. M.R. Afshar Mogaddam

E–mail addresses: [mr.afsharmogaddam@yahoo.com](mailto:mr.afsharmogaddam@yahoo.com); [Afsharmogaddam@tbzmed.ac.ir](mailto:Afsharmogaddam@tbzmed.ac.ir)

*Corresponding author: Prof. M. Torbati

E–mail address: Torbatima@yahoo.com

Tel.: +98 41 31772354

Table S1. Mobile phase composition used for separation of target AFs.

|  | Mobile phase composition (v: v: v) | | |
| --- | --- | --- | --- |
| Time (min) | Methanol | ACN | 1% v/v acetic acid in water |
| 0 to 4 held for 1min | 15 | 15 | 70 |
| 4 to 9 min and held for 2 min | 25 | 45 | 30 |


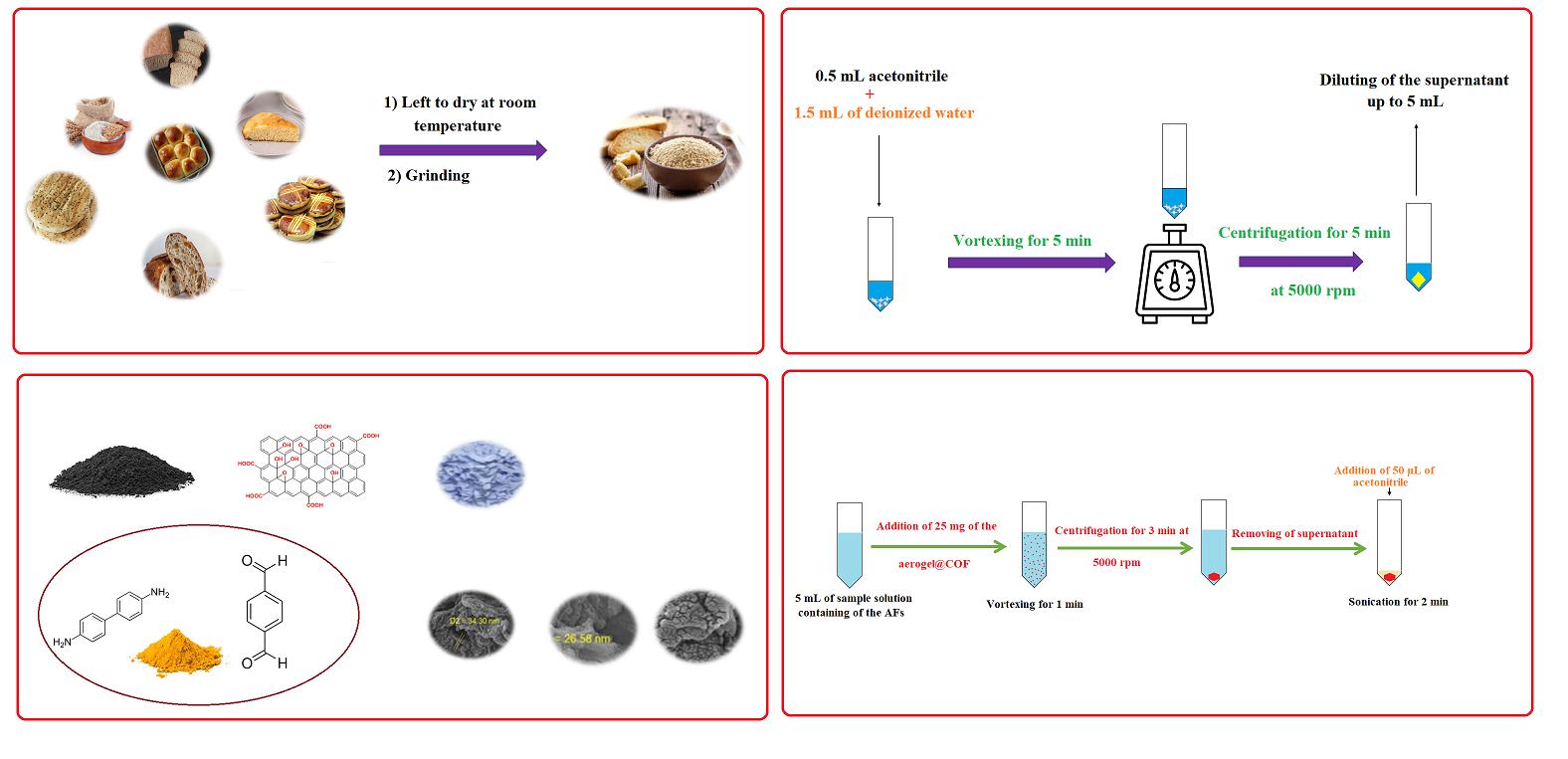


Fig. S1. Extraction method steps.


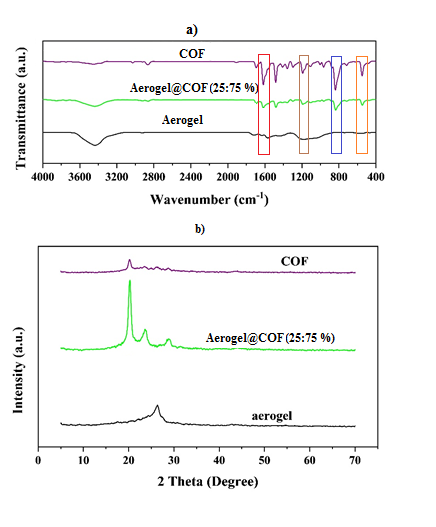


Fig. S2. (a) FTIR spectra of the aerogel, aerogel@COF (25: 75, %), and COF, and (b) XRD patterns of aerogel, (25: 75, %), and COF.


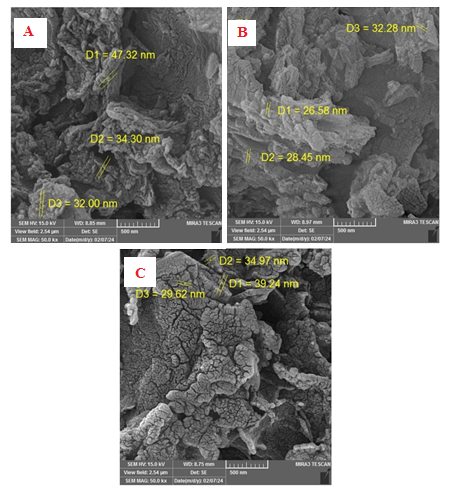


Fig. S3. SEM images of the aerogel, aerogel@COF (25:75, %), and COF.


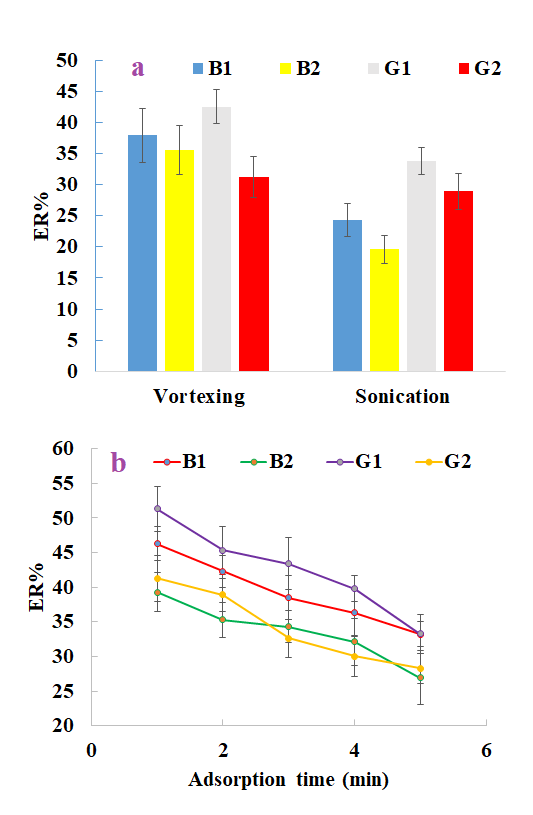


Fig. S4. Selection of agitation kind and time in adsorption step.

(a) Conditions: sample, 5 mL deionized water spiked with AFs at a concentration of 5 ng/mL; sorbent amount, 10 mg; agitation time in adsorption step, 2 min; elution solvent type (volume), ACN (50 μL); and stirring type (time) in desorption step, vortexing (2 min).

(b) Conditions: are the same as those used in Fig. S4a, except vortexing was used as the agitator in adsorption step.


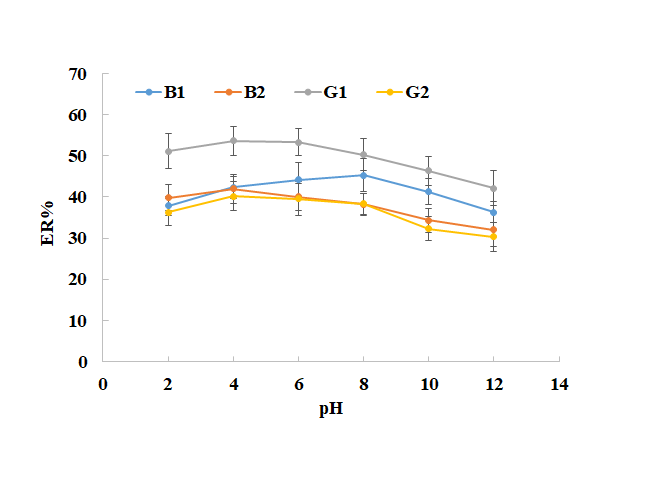


Fig. S5. pH effect.

Conditions: are the same as those used in Fig. S4b, 1 min was chosen as the optimal vortexing time.


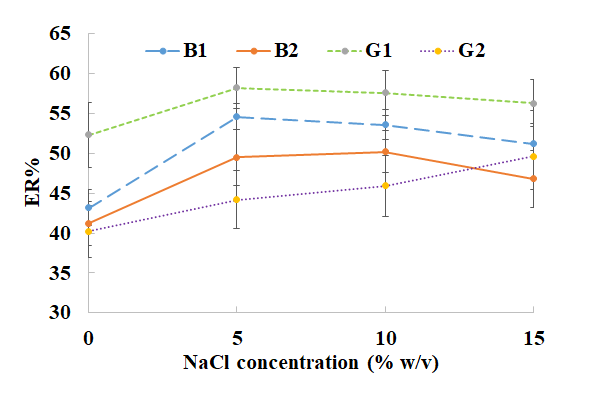


Fig. S6. NaCl addition effect.

Conditions: are the same as those used in Fig. S5a and the solution pH was not adjusted.


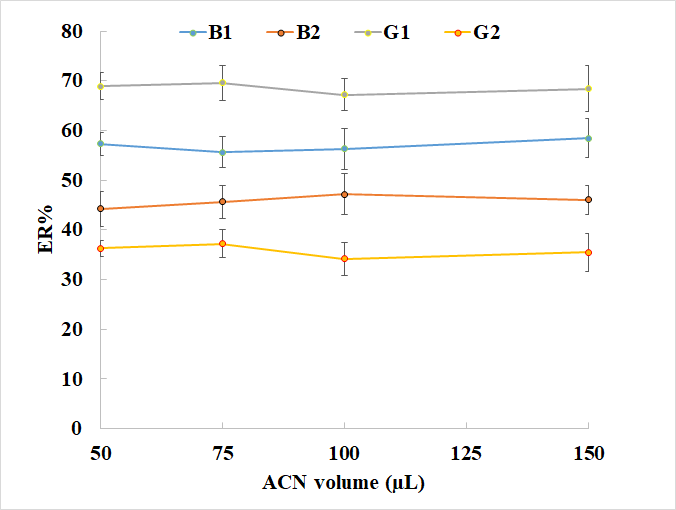


Fig. S7. Optimization of ACN volume.

Conditions: are the same as those used in Fig. S6, except the aqueous phase was used without pH adjustment.


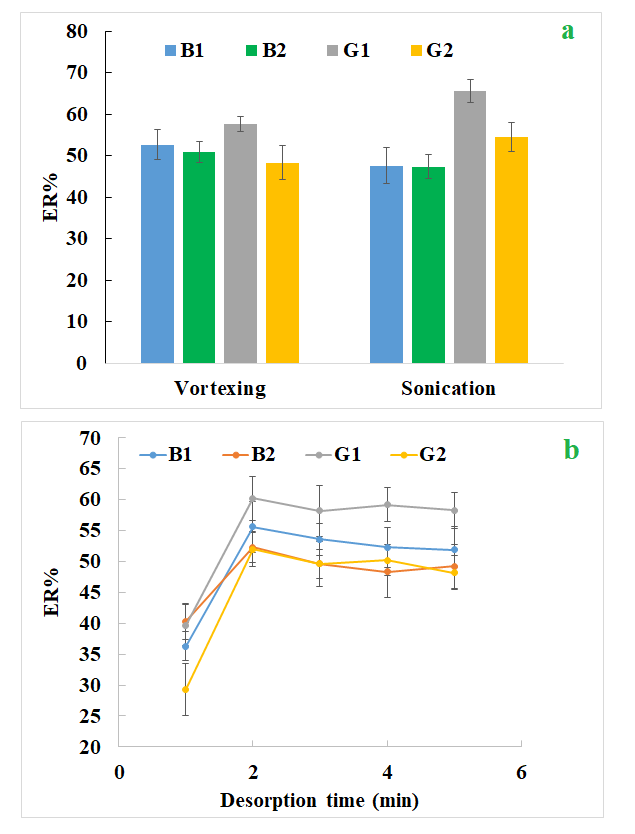


Fig. S8. Selection of agitation type and time in desorption step.

(a) Conditions: are the same as those used in Fig. S7, except 50 µL of ACN was used as the eluent.

(b) Conditions: are the same as those used in Fig. S8a, the analytes elution from the sorbent surface was done under sonication.


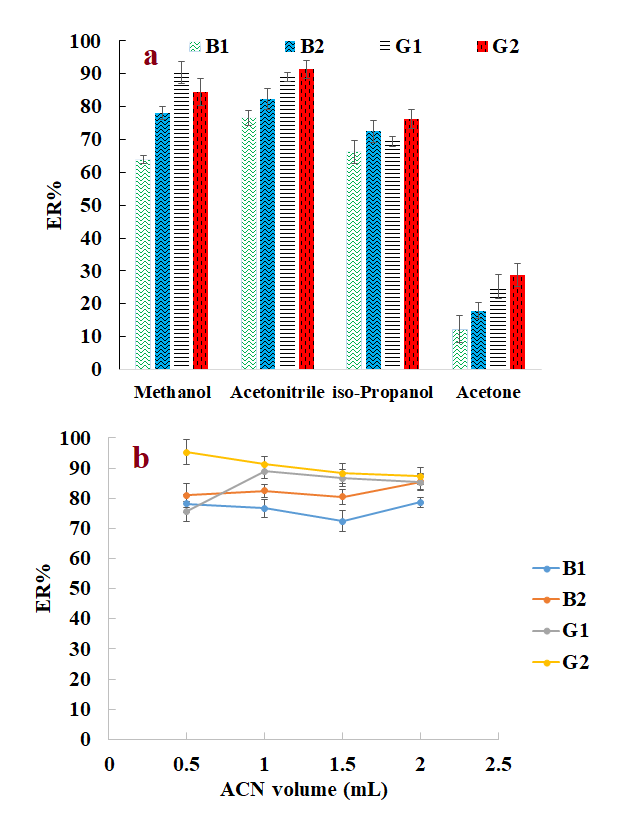


Fig. S9. Optimization of aqueous solution composition and volume in extraction of AFs from cereal samples.

(a) Conditions: are the same as those used in Fig. S8b, except 2 min was chosen as the optimal desorption time.

(a) Conditions: are the same as those used in Fig. S9a, ACN was used as the organic solvent in the extract composition

| Analyte | LOD ^a)^ | LOQ ^b)^ | LR ^c)^ | r^2^ ^d)^ | RSD % ^e)^ | | | | ER ± SD ^f)^ |
| --- | --- | --- | --- | --- | --- | --- | --- | --- | --- |
|  |  |  |  |  | Intra–day precision at a concentration of | | Inter–day precision at a concentration of | |  |
|  |  |  |  |  | 2 µg/kg | 5 µg/kg | 2 µg/kg | 5 µg/kg |  |
| AF B1 | 0.014 | 0.047 | 0.047–50 | 0.999 | 6.8 | 4.4 | 8.2 | 5.7 | 47 ± 2 |
| AF B2 | 0.009 | 0.032 | 0.032–50 | 0.996 | 5.9 | 5.5 | 7.6 | 6.8 | 43 ± 7 |
| AF G1 | 0.021 | 0.064 | 0.064–50 | 0.999 | 8.05 | 5.5 | 10.1 | 6.9 | 53 ± 5 |
| AF G2 | 0.012 | 0.039 | 0.039–50 | 0.987 | 8.3 | 6.9 | 10.2 | 8.7 | 42 ± 4 |

Table S2. Figures of merit of the developed method for determination of investigated analytes.

a) Limit of detection (S/N=3) (µg/kg).

b) Limit of quantification (S/N=10) (µg/kg).

c) Linear range (µg/kg).

d) Coefficient of determination.

e) Relative standard deviation for intra–and inter–day (n = 6) precisions.

f) Extraction recovery ± standard deviation (n=3).

| Method | Sample | LOD ^a)^ | LOQ ^b)^ | LR ^c)^ | RSD % ^d)^ | Reference |
| --- | --- | --- | --- | --- | --- | --- |
| DSPME–HPLC–FLD ^e)^ | Milk | 0.003–0.005  (µg/L) | 0.01–0.02  (µg/L) | 0.01-1  (µg/L) | <17.3 | (Shuib & Saad, 2022) |
| MDSPE-HPLC-FLD ^f)^ | Pistachio | 0.02-0.07  (µg/kg) | 0.06-0.21  (µg/kg) | _ | <7.3 | (Karami-Osboo, Ahmadpoor, Nasrollahzadeh, & Maham, 2022) |
| IL-DLLME- HPLC–FLD ^g)^ | Wheat | 0.01–0.2  (µg/kg) | 0.04-0.7  (µg/kg) | _ | 0.8-4.7 | (Zhao, An, Sun, He, Jiang, & Zhang, 2021) |
| SPE-Chip-MS ^h)^ | Peanut oil | 0.013-0.067  (µg/L) | 0.044–0.22  (µg/L) | 1.25-100  (µg/L) | <18.5 | (Chen, Liu, Li, Tan, Zhang, & Xu, 2021) |
| DSPE–HPLC–FLD | Cereal samples | 0.009–0.021  (µg/kg) | 0.032–0.064  (µg/kg) | 0.064–50  (µg/kg) | <11 | This work |

Table S3. Comparison of the proposed method with the previous ones.

a) Limit of detection

b) Limit of quantification

c) Linear range

d) Relative standard deviation

e) Dispersive solid phase microextraction–high performance liquid chromatography– fluorescence detector

f) Magnetic solid phase extraction–high performance liquid chromatography– fluorescence detector

g) Ionic liquid-based-dispersive liquid-liquid microextraction-high performance liquid chromatography-fluorescence detector

h) Solid phase extraction-based microfluidic chip-mass spectrometry

**References**

Chen, J., Liu, F., Li, Z., Tan, L., Zhang, M., & Xu, D. (2021). Solid phase extraction based microfluidic chip coupled with mass spectrometry for rapid determination of aflatoxins in peanut oil. *Microchemical Journal, 167*, 106298.

Karami-Osboo, R., Ahmadpoor, F., Nasrollahzadeh, M., & Maham, M. (2022). Polydopamine-coated magnetic Spirulina nanocomposite for efficient magnetic dispersive solid-phase extraction of aflatoxins in pistachio. *Food Chemistry, 377*, 131967.

Shuib, N. S., & Saad, B. (2022). In-syringe dispersive micro-solid phase extraction method for the HPLC-fluorescence determination of aflatoxins in milk. *Food Control, 132*, 108510.

Zhao, R., An, J., Sun, Y., He, L., Jiang, X., & Zhang, S. (2021). A simple and low-cost sample preparation for the effective extraction, purification and enrichment of aflatoxins in wheat by combining with ionic liquid-based dispersive liquid–liquid microextraction. *Microchemical Journal, 164*, 106036.
